# Improving Ultra-Low Temperature Preservation Technologies of Soybean Pollen for Off-Season and Off-Site Hybridization

**DOI:** 10.3389/fpls.2022.920522

**Published:** 2022-06-30

**Authors:** Hongchang Jia, Xin Liang, Lixin Zhang, Jinmei Zhang, Enoch Sapey, Xianyuan Liu, Yanhui Sun, Shi Sun, Hongrui Yan, Wencheng Lu, Tianfu Han

**Affiliations:** ^1^Institute of Crop Sciences, Chinese Academy of Agricultural Sciences, Beijing, China; ^2^Heihe Branch, Heilongjiang Academy of Agricultural Sciences, Heihe, China; ^3^Council for Scientific and Industrial Research (CSIR)-Oil Palm Research Institute, Kade, Ghana

**Keywords:** soybean, pollen, ultra-low temperature preservation, germination rate, off-site hybridization

## Abstract

Preserving viable pollen is of great interest to breeders to maintain desirable germplasm for future inbreeding. Ultra-low temperature preservation of pollen is an effective and safe way for long-term storage of plant germplasm resources. In this study, we improved methods for the preservation of soybean pollen at ultra-low temperature. Soybean flowers at the initially-open stage were collected at 6–10 a.m. during the fully-bloom stage of soybean plants and were dehydrated for 10 h and then frozen and stored at −196 or −80°C. *In vitro* culture experiments showed that the viability of preserved pollen remained as high as about 90%. The off-season (local site Heihe) and off-site (Beijing, after long-distance express delivery from Heihe) hybridization verification was conducted, and no significant difference in true hybrid rate was founded between the preserved pollen and the fresh pollen. The ultra-low temperature preservation technology for soybean pollen could break the spatiotemporal limit of soybean hybridization and facilitate the development of engineered soybean breeding.

## Introduction

Artificial hybridization between varieties is still the major method for soybean breeding. As the photo-thermal sensitivity of soybean limits the adaptation of soybean varieties to a wide latitude (Liu et al., 2017), it is difficult to achieve intercrossing between varieties with the diverse geographical origin and different flowering times even under the same sowing date and location (Tyagi and Hymowitz, 2003). As a result, the range of available parents is limited and the genetic basis of bred varieties is very narrow (Polito and Luza, 1988). In the conventional breeding programs, soybean hybridization could be completed only when the flowering of selected varieties is at the same time and location. Therefore, a very short time for hybridization results in low hybridization efficiency. The availability of viable pollen is a prerequisite to facilitate breeding in many species to overcome this time-and-space difficulty (Alba et al., 2011; Gowthami et al., 2021).

At present, ultra-low temperature preservation at −80°C, in liquid nitrogen (LN, −196°C), or LN vapor phase (−150 ~ −180°C) are all effective for pollen storage (Lu and Chen, 2003; Zhang et al., 2006; Chen et al., 2013; Yang et al., 2014) and for the extended maintenance of pollen viability (Barnabás and Rajki, 1976; Akihama and Omura, 1986; Li, 1987; Ganeshan and Alexander, 1990; Hu and Guo, 1996; Shi et al., 1996; Wang et al., 2003; Cheng et al., 2007; Li et al., 2010; Zhang et al., 2017; Qiu et al., 2018; Oliveira et al., 2021). Cryo-banks of pollen have been established in Japan (Akihama and Omura, 1986), the United States (Connor and Towill, 1993), Canada (Mercier, 2009), China (Zhang et al., 2009, 2014a, 2017), and India (Engelmann, 2004).

To date, the common procedure of pollen ultra-low temperature preservation is as follows: pollen collection, dehydration, freezing, thawing, and field application (Li, 1987; Polito and Luza, 1988; Liu and Wang, 2001). Pollen collection, dehydration methods, and thawing methods directly determine the success or failure of pollen preservation (Liu and Wang, 2001; Shang et al., 2018). To achieve the ideal preservation results, attention should be paid to improving the technology for the preservation of different species and tissues.

The status of pollen for collection is an important factor for pollen preservation (He et al., 2017). The plant pollen viability varies greatly in different developmental stages, which was mainly related to the maturity of pollen (Liang et al., 1993). Marchant et al. (1992) compared the Chinese rose pollen in different open states of flowers and found that pollen of unopened flowers was less likely to be contaminated and more suitable for collection.

For pollen storage, moderate dehydration is also the key to keeping post-preservation pollen viability (Sauve et al., 2000). As temperature decreases, the water inside the cell freezes to form ice crystals, which damage cell membranes and organelles under ultra-low temperature (Towill, 1981; Liu et al., 2015). To reduce or avoid the damage, the water content of the tissues should be reduced appropriately to optimal levels before storage (Nepi et al., 2001; Pacini and Hesse, 2004). And the optimal water content of pollen for preservation at −80°C and −196°C varies among species, even cultivars. Methods for pollen dehydration include desiccant drying (Hu and Guo, 1996), drying at room temperature (Liu and Wang, 2001), drying under incandescent lamps (Jiang and Gao, 1989), freeze-vacuum drying, vacuum drying, etc. Studies have shown that dehydration at room temperature helps to maintain optimal pollen viability (Rajasekharan and Ganeshan, 1994), whereas high temperature during the dehydration process adversely affects the pollen viability (D'antonio and Quiros, 1987). Drying at room temperature and drying under incandescent lamps both achieved excellent drying results (Zhang et al., 2017).

In addition, thawing methods also influenced the pollen viability after preservation (Li, 1987; Polito and Luza, 1988; Shi et al., 1995; Sauve et al., 2000; He et al., 2017). The process of thawing, water absorption, secondary freezing, and osmotic shock of water damage the cell membrane system (Zhang et al., 2006). Thus, it is very important to improve the thawing method. The methods of thawing pollen include thawing at room temperature, thawing in a warm water bath, and thawing under running water. When the cryopreserved materials were thawed, the temperature of refreezing is −5 to −10°C (Chen, 1989). A warm water bath at 30–40°C is usually used for thawing so that the pollen quickly passes through the dangerous temperature range (Liu and Zhang, 2004).

Several studies have been reported on the storage of soybean pollen. Chen and Ding (1988) found that more than 50% of the pollen dehydrated with calcium chloride was still viable on the 5th day under low-temperature conditions (2–3°C). Gai et al. (1980) found that soybean pollen with low temperature (−3 to −4°C) and dehydration treatment retained viability of 60% for storage of 14–15 days. Perveen and Khan (2009) found that high germination and viability of soybean pollen were maintained under storage in a freezer (−20 and −30°C), and the highest germination percentage was observed under a freeze drier (−60°C). Tyagi and Hymowitz (2003) investigated the germination rate of preserved pollen from seven cultivated and five wild soybean varieties and found that the range of pollen germination rate was 17–77.8% after storage in LN of 7 days without dehydration before storage. In previous studies, the effect of a single factor on the ultra-low temperature preservation of soybean pollen was studied, however, there is a lack of systematic study. This study was undertaken to investigate the effects of pollen collection, dehydration, freezing, thawing, and hybridization verification on the ultra-low temperature preservation of soybean pollen. It is aimed to develop a simple and efficient preservation protocol and realize the breeding application of preserved pollen through long-term storage and long-distance transportation.

## Materials and Methods

### Materials

The soybean pollen used for the investigations of preservation technology was collected from a purple flower variety Heihe43, which is the most widely grown soybean variety, in the Heihe City, Heilongjiang Province, China. In the hybridization verification investigation conducted in Heihe, the white flower variety Jinyuan55 was used as the female parent and the fresh pollen was collected from Heihe43. In the hybridization validation carried out in Beijing, a local white flower variety Zhonghuang39 was used as the female parent. Considering that Heihe43, an elite variety from the north part of northeast China, grew poorly and produced a limited size and number of flowers in Beijing, the fresh pollen was taken from a local purple flower variety Zhonghuang30, an elite variety at Huang-Huai-Hai Valley, to further prove the feasibility of the off-site use of pollen preservation technologies.

### Methods

#### Planting Conditions

In Heihe, Heilongjiang province of China, the field experiments were conducted in 2018–2020 in the Experiment Station of the Heihe Branch of Heilongjiang Academy of Agricultural Sciences (50°15'N, 127°27'E). The sowing dates were May 9, May 11, and May 14, respectively in the relative years. Heihe43 and Jinyuan55 were planted in a 1.5 m row, with 0.6 m space between the rows and a space of 0.07 m between the adjacent plants. In Beijing, Zhonghuang30 and Zhonghuang39 were grown in pots at the campus of the Institute of Crop Sciences, Chinese Academy of Agricultural Sciences (40°130'N, 116°330'E). The seeds of Zhonghuang30 and Zhonghuang39 were planted on 15 June 2020 and 22 June 2020, respectively. Twenty pots for each variety and five uniform plants remained in each pot.

#### Stage Determination for Collecting Soybean Flowers Bearing Highly Viable Pollen

**Figure 1:**
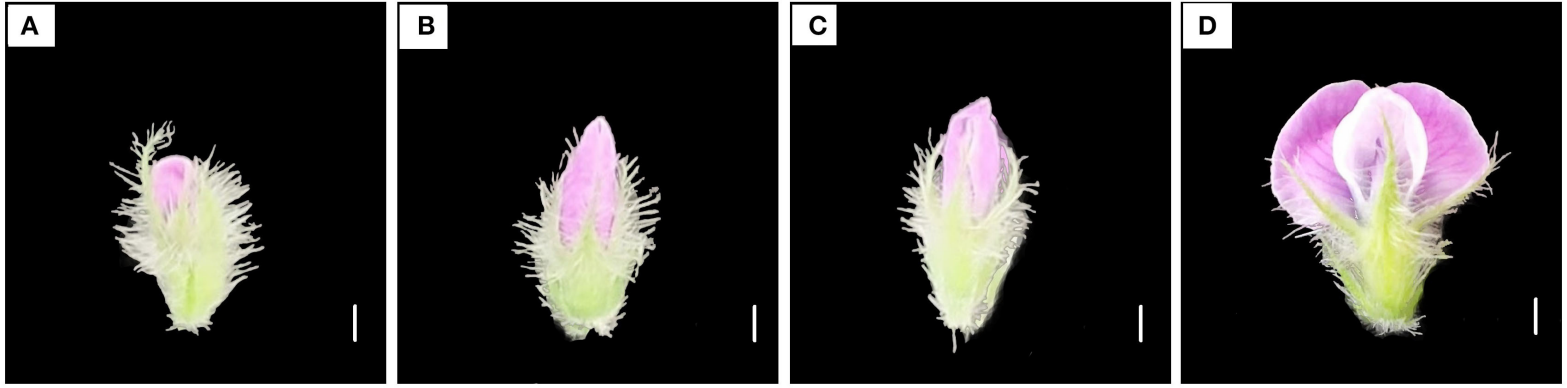
Flower characteristics at four developmental stages. **(A)** Petal-emerging stage; **(B)** Petal-elongated stage; **(C)** Initially-open stage; **(D)** Fully-open stage. Scale bar, 1 mm.

**Figure 2:**
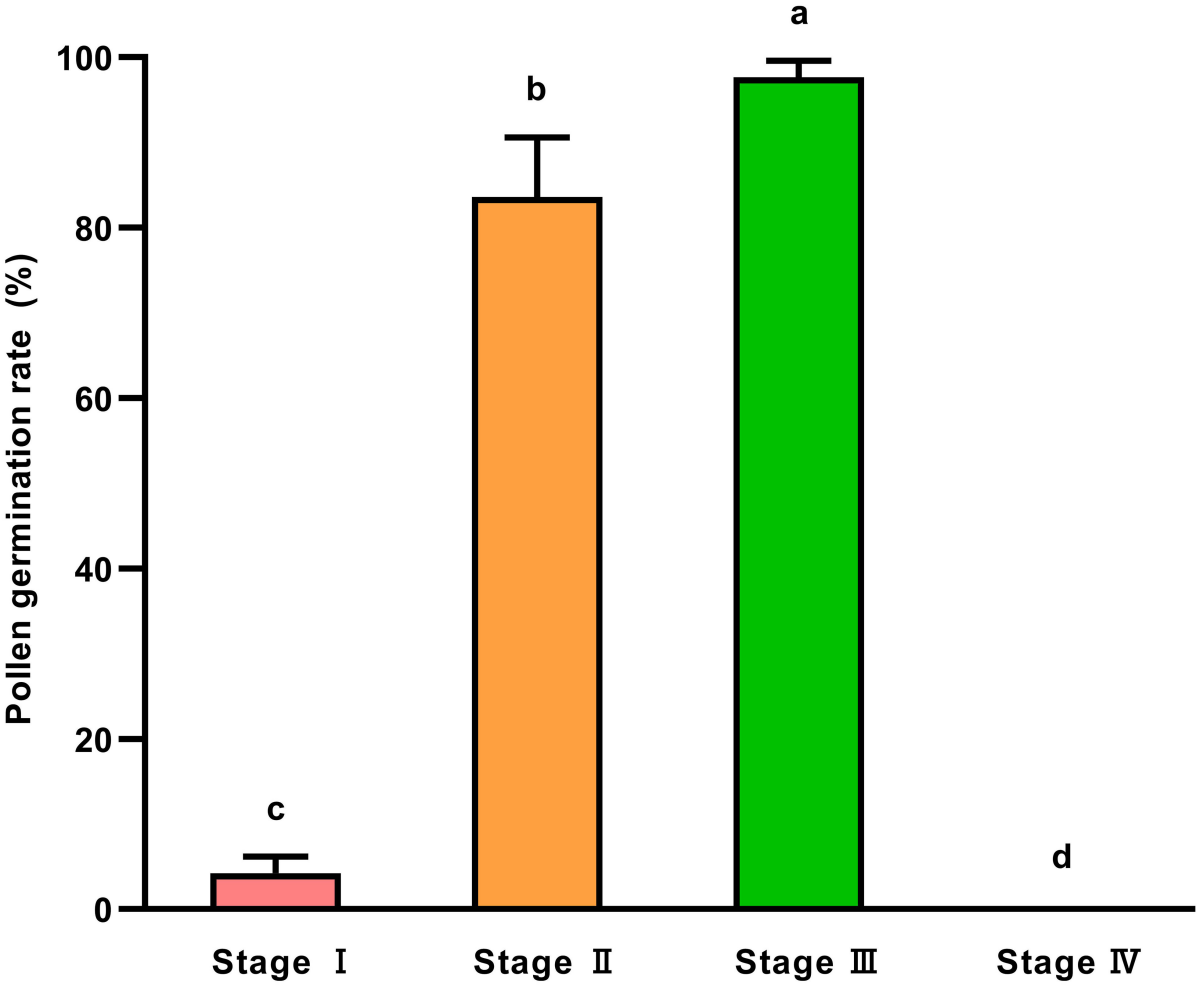
Germination rate of fresh pollen at different developmental stages of soybean flowers. Stage I: Petal-emerging stage; Stage II: Petal-elongated stage; Stage III: Initially-open stage; Stage IV: Fully-open stage. All data were represented as mean ± SD of three replicates. Welch's and Dunnett's T3 methods were used to compare the means of germination rates of fresh pollen at different developmental stages of soybean flowers (*P* < 0.05).

**Figure 3:**
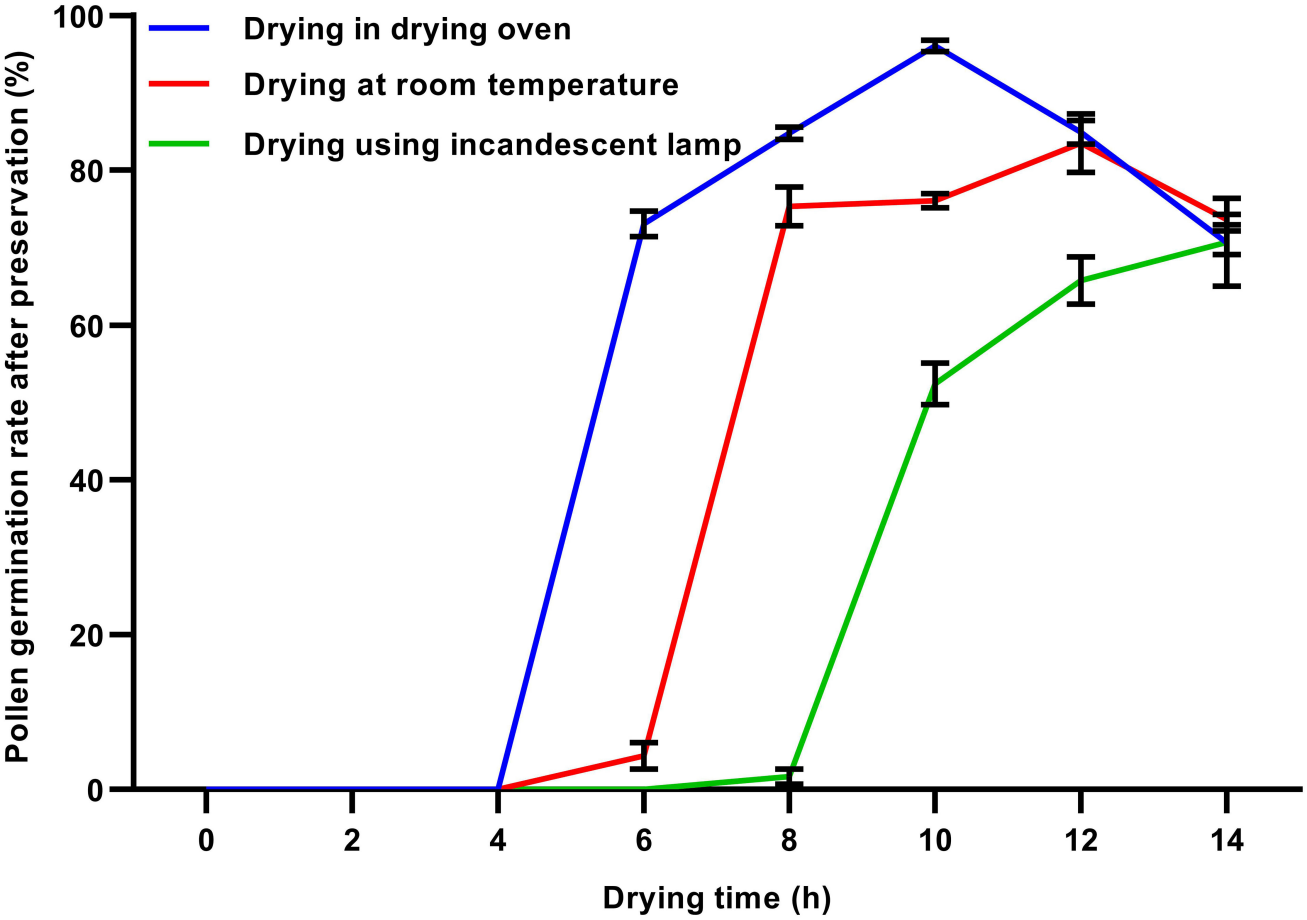
Effects of different dehydration treatments on pollen germination rates after preservation. The flowers were collected at the initially-open stage, dehydrated under three treatments at different times, frozen in liquid nitrogen (LN) for 72 h, and thawed at room temperature. All data were represented as mean ± SD of three replicates.

**Figure 4:**
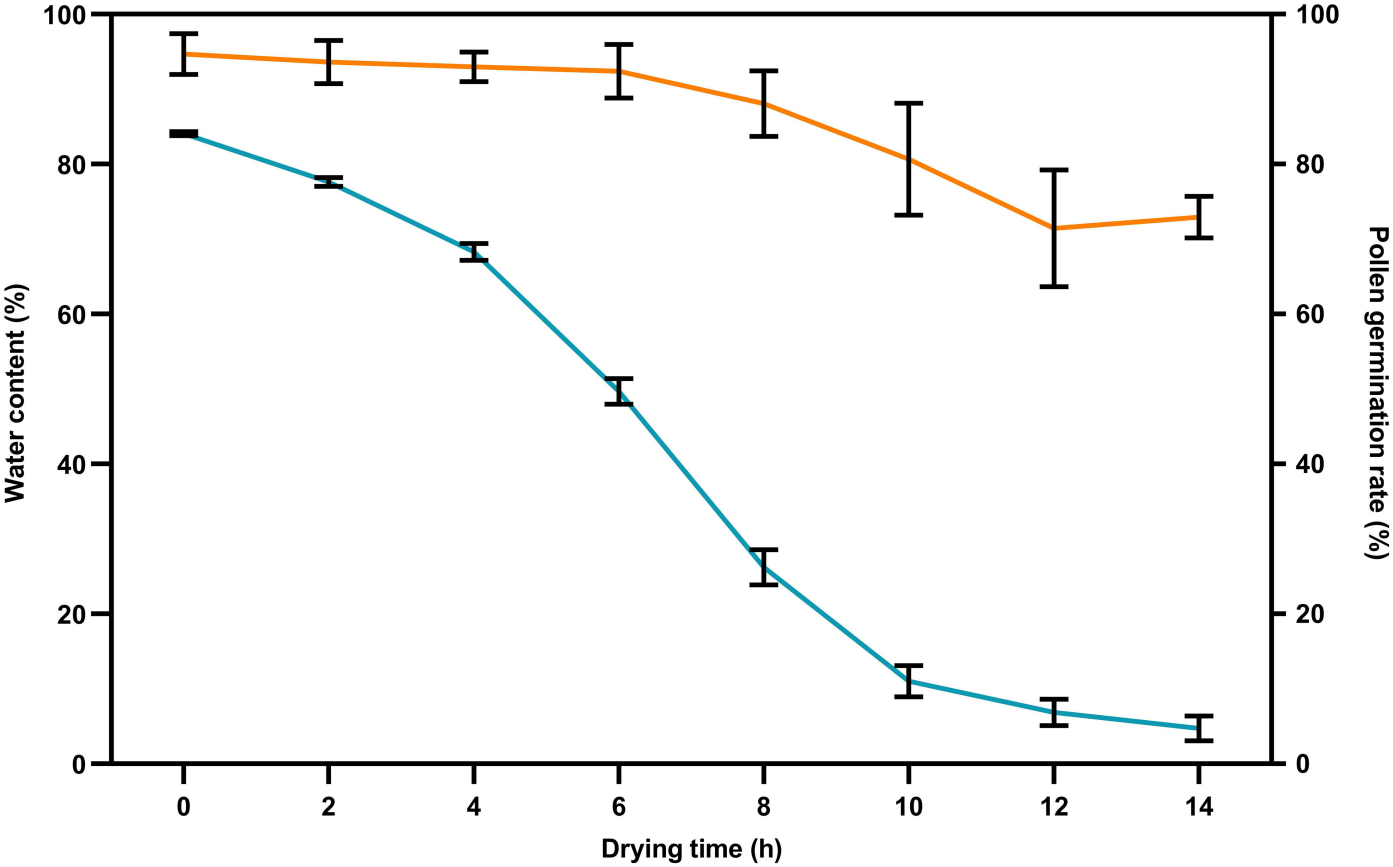
Water contents of flowers and germination rates of pollen at different drying times. The flowers of Heihe43 at the initially-open stage were collected and dehydrated in the drying oven. All data were represented as mean ± SD of three replications.

Based on the degrees of opening of the petals, soybean flowers of Heihe43 were categorized into the following four stages (Figure 1): (1) Petal-emerging stage: the petals have just emerged from the calyx and their color can be distinguished, but the height is lower than the calyx (Figure 1A); (2) Petal-elongated stage: the petals have extended and exceeded the height of calyx, but the banner flap is still closed (Figure 1B); (3) Initially-open stage: the petals grow to the maximum height, and there are obvious cracks at the top of the banner petal (Figure 1C); (4) Fully-open stage: wing and keel petals are all fully unfolded (Figure 1D). We collected the intact flowers at four stages, respectively, for the detection of pollen viability by *in vitro* culture as described in Section Detection of Pollen Viability.

#### Dehydration Treatments

The freshly collected soybean flowers were 150 g with three replications. Dehydration treatment was carried out using the oven, incandescent lamp, and natural drying, respectively. The details are as follows:

(1) Dehydrated in the oven: The flowers were placed in a plastic pallet and then were put into the electrothermal blowing drying oven (Lichen101-3BS) for continuous dehydration for 14 h. The temperature was set at 35°C and the humidity in the oven was adjusted to 25% by silica gel desiccants. The ratio of the flower and silica gel was about 1:7. The temperature and humidity in the drying oven were monitored with a temperature and hygrometer, respectively.

(2) Dehydrated using incandescent lamps: The flowers were laid on sulfate paper and placed under a 40 W incandescent lamp for 14 h at room temperature of 25°C and humidity of 55%. The lamps were 15 cm above the flowers, and the light intensity was about 10 μmol m^−2^ s.

(3) Dehydrated at room temperature: The flowers were spread on sulfate paper and placed indoors for 14 h at room temperature of 25°C and humidity of 55%.

During the dehydration process, flowers were randomly taken from each of the above three treatments at drying intervals of 2 h, i.e., 0, 2, 4, 6, 8, 10, 12, and 14 h, respectively, and then the measurement of pollen germination rate was conducted by *in vitro* culture as described as in Section Detection of Pollen Viability. Other dehydrated flowers were preserved and used in subsequent experiments.

#### Determination of Water Content of Soybean Flowers

Before dehydration, the samples were divided into six parts with three repeats, and about 2 g of each sample was weighed by an analytical balance (ZX224ZH), placed in the Petri dishes, and dehydrated in the drying oven as described in Section Dehydration Treatments. The value of flower weight (FW_i_) was recorded at drying intervals of 2 h, i.e., 0, 2, 4, 6, 8, 10, 12, and 14 h drying, respectively. The dry weight (DW) was recorded when it was dehydrated to constant weight in a drying oven at 110°C. The water content (WC_i_, %) was computed using the following formula:


$$\begin{array}{l} WC_{\text{i}}=\frac{FW_{i}-DW}{FW_{i}}\times 100\% \end{array}$$

WC_i_ (%) represents the water content of the flower at i h, FW_i_ represents the flower weight at i h, and DW represents the final DW.

#### Freezing and Preservation Methods of Soybean Flowers

Flowers were taken from the drying oven with a temperature of 35°C and humidity of 25% at intervals of 2 h, and each sample was about 1 g in weight, with three replications. The samples were wrapped with the tin foil tightly and then followed by three freezing and preservation treatments: (1) Freezing and preserving in LN; (2) Freezing and preserving in a −80°C freezer (Haier DW-86L338); (3) Freezing in LN and then transferring to a −80°C freezer: put flowers in LN for 72 h and then transfer them to −80°C freezer to preserve. After 1 week, the flower was taken out, thawed, and the pollen germination rate was assessed by *in vitro* culture as described in Section Detection of Pollen Viability.

#### Thawing Methods of Soybean Flowers

After taking out the tin foil package and wrapping the flowers from the preservation condition described in Section Freezing and Preservation Methods of Soybean Flowers, the samples in the package were thawed for 1 min with the following three methods, respectively: (1) thawing under running water at 20°C (±5°C); (2) the package was put in tubes and thawed in the water bath of 35°C; and (3) thawing at room temperature of 25°C (±2°C). The experiment was repeated three times, with six flowers for each repeat selected to measure pollen germination rates by *in vitro* culture as described in Section Detection of Pollen Viability.

#### Detection of Pollen Viability

##### In vitro Culture

The culture medium was prepared using the methods reported by Wang et al. (2016) with some modifications: 19.2% (wt%) sucrose, 68.9 mg /L GA_3_, 0.015% (wt%) H_3_BO_3_, 0.05% (wt%) CaCl_2_, 7.5% (wt%) PEG-4000, and the solvent was water. The pollen was cultured in the *in vitro* medium for 20 min at room temperature (25±2°C). The average pollen germination rate was observed under microscopes (Murzider-D106B) randomly and calculated using the data from six fields of six flowers (one for each flower) with three replications. Pollen was scored as germinated if the length of the pollen tube was over two times its diameter, using the formula as indicated below:


$$GR=\frac{b}{a}\times 100\%$$

GR: Germination rate; *a*: the total number of pollen in each field under microscopes; *b*: the number of the germinated pollen in each field under microscopes.

##### Hybridization Verification

Off-season hybridization: In 2018, the flowers of Heihe43 at the initially-open stage were collected in Heihe, dehydrated for 10 h in the drying oven, wrapped with tin foil, frozen in LN for 72 h, and then transferred to a −80°C freezer for 1 year from 15 July 2018 to 24 July 2019. The off-season hybridization was conducted in July 2019 in Heihe. The preserved pollen was taken out in batches from the freezer and thawed at room temperature, and the white flower variety Jinyuan55 (female parent) was pollinated with the preserved pollen. Freshly collected flower at the initially-open stage from Heihe43 in 2019 was used as control and pollination was made synchronously.

Off-site hybridization: On 15 July 2020, flowers of Heihe43 at the initially-open stage were collected in Heihe, dehydrated for 10 h and 14 h in the oven, and were wrapped and frozen in LN for 72 h, and then transferred to a −80°C freezer. After 31 days of preservation, the samples were placed inside a rigid foam plastic box containing 15 kg of dry ice and sent to Beijing (over 1,800 km away from Heihe) by express delivery. After 3 days, it arrived in Beijing and was stored in the −80°C freezer. From 21 to 25 August 2020, the preserved pollen was taken out in batches from the freezer and thawed at room temperature before use. Pollination was carried out using Zhonghuang39 as the female parent. Fresh pollen of purple flower variety Zhonghuang30 was used as control and pollinated synchronously.

Among the F1 individuals, the plants with purple flowers were regarded as true hybrids and those with white flowers were regarded as false hybrids. The true hybrid rate was calculated by dividing the number of true hybrids by the total number of hybrid plants.

#### Statistical Analysis

The IBM SPSS 19 S Statistics software was used to calculate the descriptive statistics, including the test for homogeneity of variance and one-way ANOVA. Means were separated by Duncan's multiple range test at *P* < 0.05, where the *F*-test was significant. Welch's and Dunnett's T3 methods were used to compare the means of different treatments for unequal variances.

## Results

### Germination Rate Differences of Fresh Soybean Pollen at Different Developmental Stages of Flowers

We collected intact flowers at four stages, respectively, for the detection of pollen viability by *in vitro* culture as described in Section Detection of Pollen Viability. The results showed that the average germination rate of fresh pollen collected from intact flowers of Heihe43 was as low as 4.26% at the petal-emerging stage, reaching 83.5 and 97.6% at petal-elongated and initially-open stages, respectively, and failed to germinate during fully-open stage (Figure 2). It suggested that pollen was highly viable during initially-open stages but rapidly declined after the flower fully opened.

### Effects of Dehydration Treatments on Soybean Pollen Viability After Ultra-Low Temperature Preservation

To verify the effects of different dehydration methods on pollen viability after ultra-low temperature preservation, the fresh pollen was dehydrated, respectively, in the oven, at room temperature, and under incandescent lamps before preservation. The results showed that within 4 h of dehydration, the dehydrated pollen in three dehydration treatments failed to germinate after preservation in LN (Figure 3). When the dehydration time was 6 h, the germination rates of soybean pollen dehydrated in an oven, at room temperature, and under an incandescent lamp were 70.1, 4.9, and 0%, respectively, after preservation in LN (Figure 3). With the extension of drying time, the post-preservation pollen germination rates in three dehydration treatments showed an upward trend. The highest post-preservation pollen germination rates in three pre-freezing dehydration treatments were found in the oven drying for 10 h (96.04%), at room temperature for 12 h (83.26%), and under an incandescent lamp for 14 h (70.72%), respectively. Drying in an oven was the fastest way to achieve the high post-preservation pollen germination rate among three dehydration methods.

Low-water content was a precondition for ultra-low temperature preservation of pollen. In the current study, the water content of freshly collected soybean flowers was as high as 84.49% (Figure 4). With the extension of drying time in the oven, the water content of the flower dropped to 41.65% for 6 h, 21.28% for 8 h, 11.0% for 10 h, 6.86% for 12 h, and 4.72% at 14 h (Figure 4). The average germination rate of soybean pollen dehydrated in the oven was above 90% within 6 h, 80.68% at 10 h, and 72.93% at 14 h (Figure 4), indicating that the 10-h dehydration in the oven could reduce the water content of pollen to an optimal range for ultra-low temperature preservation.

### Effects of Freezing and Preservation Methods on Germination Rates of Preserved Pollen

**Figure 5:**
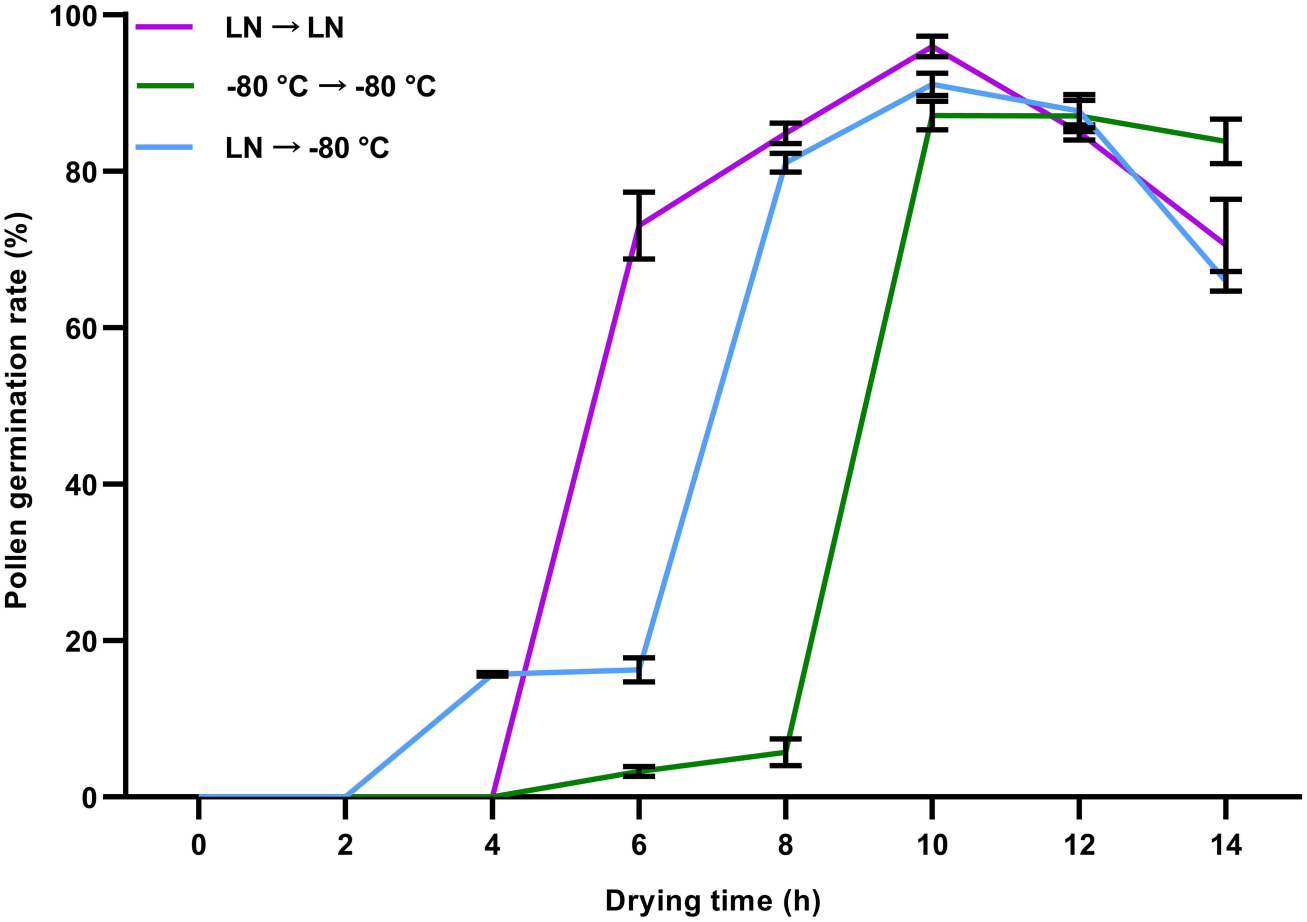
Germination rates of soybean pollen after different freezing and preservation treatments. LN → −80°C, flowers were frozen in liquid nitrogen (LN) for 72 h and then stored in a −80°C freezer; −80°C → −80°C, flowers were frozen and preserved in a −80°C freezer; LN → LN, flowers were frozen and preserved in LN. The flowers at the initially-open stage were collected and dehydrated in a drying oven at different times. After preservation, the flowers were thawed at room temperature for germination tests. All data were represented as mean ± SD of three replicates.

To determine the suitable ultra-low temperature preservation methods, the influence of freezing and preservation methods on pollen viability was investigated. The post-preservation germination rate of the pollen dehydrated in the oven was detected under three freezing and preservation treatments, including (1) frozen and preserved in LN; (2) frozen and preserved in −80°C freezer; and (3) frozen in LN for 72 h and then preserved in −80°C freezer. The results showed that within 4 h of dehydration, the average post-preservation germination rate of pollen frozen in LN and then stored in a −80°C freezer was <20%, whereas those of pollen frozen and stored in LN and those frozen and stored in a −80°C freezer were both 0 (Figure 5). When the drying time was 6 h, the post-preservation germination rate of pollen frozen in LN and then preserved in a −80°C freezer was 16.26%, those which were frozen and preserved in LN was 73.04%, and those which were frozen and preserved in a −80°C freezer was 3.26%. When the drying time was 10 h, the average post-preservation pollen germination rate of all three treatments reached the peak, at about 87.08% (frozen and preserved in a −80°C freezer), 91.08% (frozen in LN for 72 h and then preserved in a −80°C freezer), and 95.95% (frozen and preserved in LN), respectively (Figure 5). The germination rate of preserved pollen with dehydration for 12–14 h showed a decreasing trend but was still higher than 70%, which could meet the demand for soybean hybridization.

### Effects of Thawing Methods on Germination Rates of Preserved Pollen

**Figure 6:**
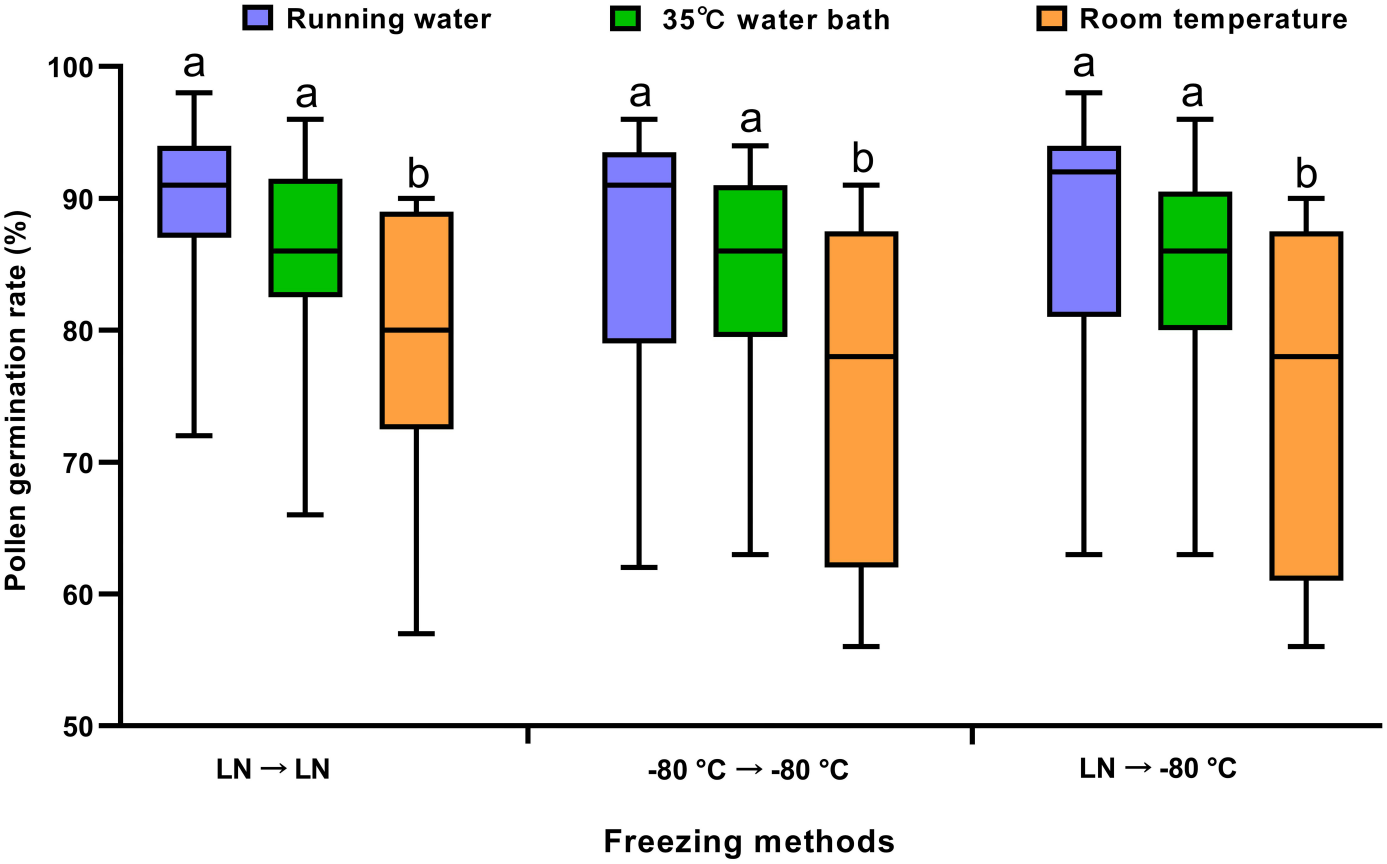
Comparison of germination rates of preserved soybean pollen after different preservation and thawing methods. LN → −80°C, frozen in liquid nitrogen for 72 h and then stored in a −80°C freezer; −80°C → −80°C, frozen and preserved continuously in a −80°C freezer; LN → LN, frozen and preserved in liquid nitrogen. The preserved samples were thawed for 1 min by three methods. Means of germination rates of preserved pollen after different preservation and thawing methods were separated by Duncan's multiple range test at *P* < 0.05, where the *F*-test was significant.

To select the suitable thawing method for soybean pollen after ultra-low temperature preservation, the flower samples wrapped with the tin foil packages were thawed in the following three methods: thawing in a water bath at 35°C, at room temperature, and under running water, respectively. The results showed that the germination rates of preserved pollen thawed under the running water and in a 35°C water bath were significantly higher than those thawed at room temperature (*p* < 0.05) regardless of the freezing and preservation methods (Figure 6). Even that, the pollen germination rates by thawing at room temperature was over 75%, which was high enough for hybridization.

### Application of Preserved Pollen in Soybean Hybridization

#### Off-Season Hybridization Application of Preserved Pollen in Heihe

**Figure 7:**
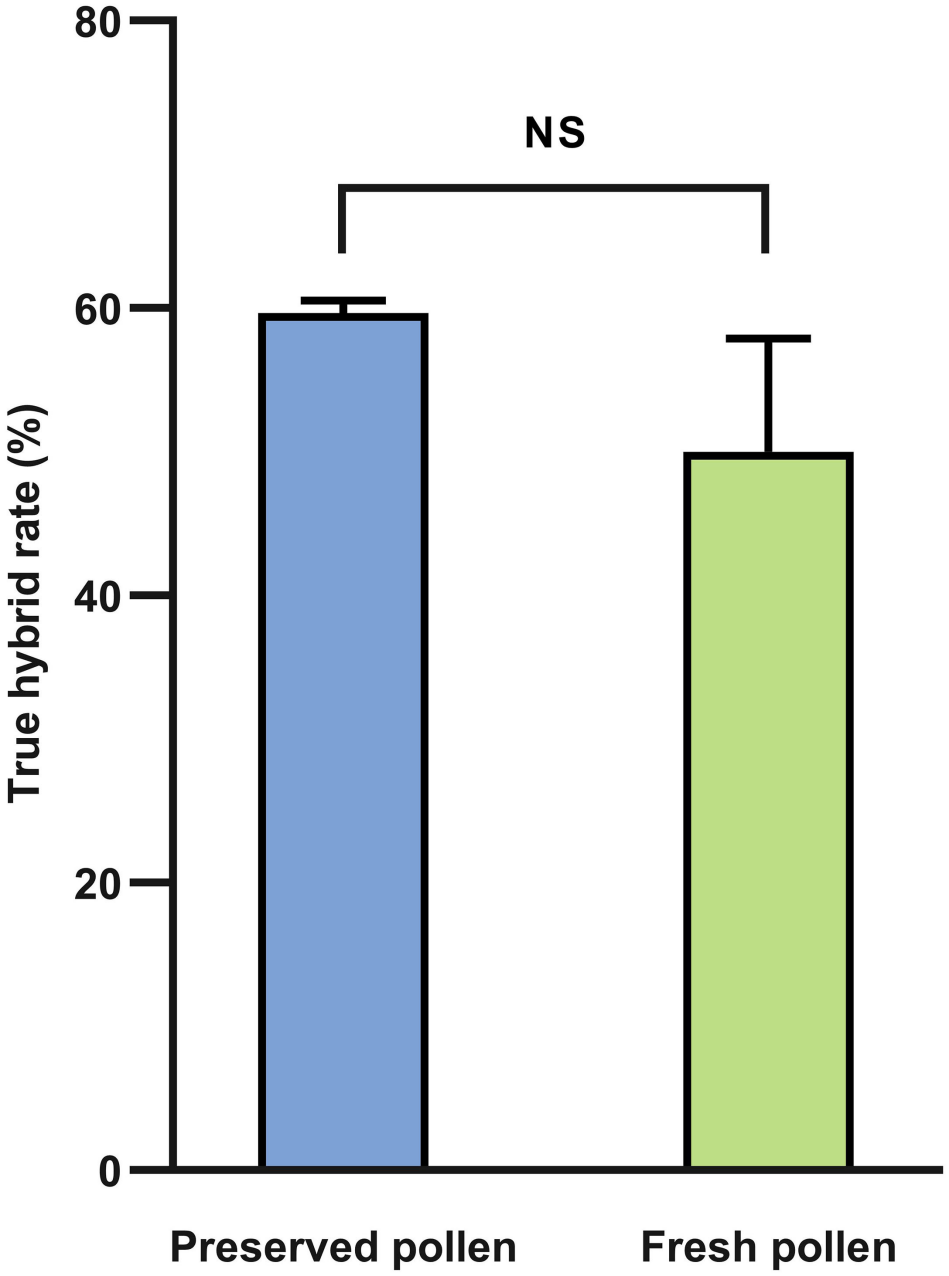
Comparison of the true hybrid rates in F1 generation of combinations using preserved and freshly collected flowers as the pollen sources. Preserved pollen: the flowers of a purple flower variety Heihe43 at the initially-open stage were collected, dehydrated, and frozen in liquid nitrogen (LN) for 72 h and then stored at −80°C, from 15 July 2018 to 24 July 2019; Fresh pollen: Fresh flowers of Heihe 43 at the initially-open stage was collected in Heihe within 0.5 h to pollinate before hybridization. Data were represented as mean ± SD of two replicates. Means of the true hybrid rates in F1 generation of combinations using preserved and freshly collected flowers as the pollen sources were separated by Duncan's multiple range test at *P* < 0.05, where the *F*-test was significant.

To verify the viability of 1-year preserved pollen of Heihe43 at −80°C, the hybridization experiment was conducted in 2019. Both the preserved and fresh pollen were used to pollinate Jinyuan55 as the female parent. The results showed that there was no significant difference in the true hybrid rate of F1 generation between being pollinated with the preserved pollen for 1 year and the fresh pollen (Figure 7), demonstrating that ultra-low temperature preservation well maintained the viability of the soybean pollen.

#### Off-Site Hybridization Application of Preserved Pollen in Beijing

To test the feasibility of the use of preserved pollen in other regions, the preserved pollen (flowers) was transported to Beijing, which is 1,800 km away from Heihe, and the hybridization verification tests were carried out in Beijing in July 2020. The results showed that the pollination with preserved pollen resulted in an even higher true hybridization rate than that using fresh pollen of local elite variety Zhonghuang30 (Table 1).

**Table 1:** Effects of preserved pollen on off-site pollination in Beijing.

**Female parent**		**Male parent**			**Pre-treatment of flowers**	**Number of plants**	**True hybrid number**	**True hybrid rate (%)**
**Variety**	**Site**	**Variety**	**Pollen collection site**	**Pollen collection date**				
Zhonghuang39	Beijing	Heihe43	Heihe	15th July	Dehydrated for 10 h	30	27	84.4
Zhonghuang39	Beijing	Heihe43	Heihe	15th July	Dehydrated for 14 h	15	14	93.3
Zhonghuang39	Beijing	Zhonghuang30	Beijing	21st August to 25th August	No (fresh)	5	3	60.0

*On 15 July 2020, pollen of Heihe 43 at the initially-open stage was collected, dehydrated, frozen in LN for 72 h, and then stored at −80°C. On 16 August 2020, the package was sent to Beijing by express delivery. From 21–25 August 2020, hybridization verification tests were carried out in Beijing using the preserved pollen sent from Heihe by express delivery*.

## Discussion

### Optimal Developmental Stages of Soybean Flowers for Pollen Collection

Pollen germination rate varies at different developmental stages of flowers in plants. For *Ziziphus* moss, the germination rate of nearly and fully mature pollen was higher than young pollen (Liang et al., 1993). For dogwood (*Cornus florida*), pollen collected from flowers with dehiscence anther was suitable to store (Sauve et al., 2000). In this study, the viability of pollen collected from the flowers at the initially-open stage was higher (Figure 2). The appropriate time for collecting soybean flowers at Heihe was 6–10 a.m. The morphological characteristics of soybean flowers that bore high-viability pollen were determined, ensuring the sampling consistency and high germination rate of pollen. In addition, collecting intact soybean flowers avoided pollen contamination, improved the efficiency of pollen collection, and facilitated the large-amount preservation of pollen at ultra-low temperature.

### Selection of Dehydration, Freezing, Preservation, and Thawing Methods for Soybean Pollen

The survival of plant cells after freezing and ultra-low temperature preservation is dependent on the lethal effects of intracellular ice. It is necessary to reduce the water content of pollen to prevent cell damage. Optimal water content is a major factor and varies among species (Zhang et al., 2006; Zhou et al., 2013; Liu et al., 2015; Yu et al., 2019). For litchi pollen, the suitable dehydration method was the use of an air-blowing electric dryer at 35°C for 6 h (Wang et al., 2015). In this study, the water content of freshly collected soybean flowers was around 84% (Figure 4), and the pollen hardly germinated when directly stored in LN without dehydration. It was found that soybean pollen dehydrated in the drying oven presented germination capacity better than at room temperature and under the incandescent lamp, and 10 h was the optimal drying time for preservation of pollen (Figure 3).

Besides dehydration, pollen viability can be affected by cooling speed, i.e., slow vs. fast cooling, during the preservation procedure (Dinato et al., 2020). A combination of fast-drying and cooling rates may enable the survival of pollen due to the reduction of the time of exposure to dehydration-related deleterious biochemical changes and the inhibition of intracellular ice-crystal formation (Impe et al., 2022). In this study, the 10 h-dehydrated pollen in the drying oven was suitable for all these three freezing and preservation methods, and the 8 h-dehydrated pollen in the drying oven was suitable for preservation in LN, and also suitable for freezing in LN and then preservation in a −80°C freezer (Figure 5). For preservation in LN, it is necessary to maintain the stable LN supply and special container, while the −80°C preservation can be conducted in a −80°C freezer. The preference of freezing and preservation at −196°C, freezing at −196°C, and preservation at −80°C would be up to the preservation conditions available and the ultimate goals for preservation.

Optimal thawing treatments are to avoid ice crystallization in the cells and to prevent the osmotic shock of water during thawing and water absorption from damaging the cell membrane system (Zhang et al., 2006). The suitable temperature range for ice crystallization is −3 ~ −50°C (Liu et al., 2015). If the temperature rises slowly during the thawing treatment and the pollen is in the “dangerous temperature zone” for a long time, the free water will recrystallize, which damages the cells and reduces the pollen vitality. Wang et al. (2003) found that thawing in a gradual treatment of −20°C (12 h) → 4°C (12 h) → 25°C (12 h) had better potato pollen viability than thawing in a continuous 35–40°C water bath. The thawing effect in running water and warm water bath had no differences and were better than at room temperature (Zhang et al., 2006). This is consistent with our results. Although the viability of pollen thawing at room temperature is slightly lower, it also meets the pollination requirements. Therefore, we selected the thawing method according to the specific situation in breeding.

### Off-Season and Off-Site Applications of Preserved Soybean Pollen in Breeding

Pollination is the most direct and effective method to evaluate the viability of preserved pollen (Liu et al., 2015). In this study, the hybridization experiments were carried out locally but off-season in Heihe and off-sited in Beijing, respectively. Hybridization results are greatly affected by many factors, such as temperature, air humidity, the expertise of the person carrying out the hybridization, etc. To ensure the reliability of the hybridization, the crossing in both Heihe and Beijing were carried out in uniform environments and conducted each by a skilled person in soybean hybridization. In Heihe, the true hybrid rate of the F1 generation of being pollinated with the preserved pollen for 1 year was as high as 59.68% (Figure 7), demonstrating that ultra-low temperature preservation maintained the viability of the soybean pollen at least for 1 year.

The maintenance of pollen viability using ultra-low temperature preservation has been realized in many species. It was reported that the viability of 8–10-years cryopreserved pollen from 12 species/cultivars of ornamental plants was higher than the fresh pollen, 17 species/cultivars retained the same viability as the control, and the viability of pecan pollen was still significantly higher than that of fresh pollen after cryopreservation (preservation in LN) of 13 years (Sparks and Yates, 2002; Ren et al., 2019). In maize, there was no significant difference in pollen pollination capacity between 1- and 2-years cryopreserved pollen and the control (Shi et al., 1996). In pecan and sweet cherry, pollen maintained over 50% viability when stored in a −80 °C freezer for 1 year (Ozcan, 2020; Wang et al., 2021). The viability of cryopreserved pollen in some species or cultivars showed a decreasing trend during the cryopreserved process (Ren et al., 2019). The differences in the storage tolerance of pollen in diverse species/cultivars may be also attributed to the pollen type, pollen size, plant taxonomy, etc., besides the pretreatments of preservation. It was known that the binucleate pollen is tolerant to dehydration and has greater viability when compared to trinucleate pollen (Dinato et al., 2020). Soybean pollen is binucleate (Albertsen and Palmer, 1979), and the pollen longevity might be longer by using the improved ultra-low temperature preservation technologies in this study. The monitoring of the preserved pollen and the stress response of preserved pollen under ultra-low temperature was investigated.

In this study, the germination rate of pollen after ultra-low temperature preservation was higher than that of pollen before freezing (Figures 4, 5), and the true hybridization rate of preserved pollen was higher than fresh pollen as well (Figure 7, Table 1). Similar findings were previously reported in other crops (van der Walt and Littlejohn, 1996; Liu et al., 2001). The reason is still not well-understood (Zhang et al., 2006, 2009; Li et al., 2010).

In the breeding programs under the field conditions, parents of a cross must synchronize during flowering time, which limits the scope of parent selection and leads to a narrow genetic base of new varieties. Soybean is a short-day plant, and the flowering time can be adjusted through artificial photoperiod treatments, sowing-date change, and other measures. However, the photoperiod treatment was costly, and under short-day conditions, soybean plants grew poorly, and produce flowers with a limited size and number of flowers (Zhang et al., 2014b), which was not conducive to pollen production and pollination. Date-of-planting was time-consuming and limited to narrow parents with similar maturity groups (Song et al., 2019). Off-season and off-site applications of preserved pollen allow the collection of flowers at any time and long-term preservation for backup, breaking through the asynchronous flowering barrier between parents belonging to different maturity groups, which will greatly broaden the range of parents for soybean breeding, expand the genetic base of new varieties, and facilitate the engineered breeding of soybean.

## Conclusion

In this study, we improved ultra-low temperature preservation technologies of soybean pollen for off-season and off-site hybridization. The procedures can be summarized as follows:

(1) Collect the intact soybean flowers at initially-open stages in the morning (6–10 a.m.).

(2) Dry the soybean flowers in a drying oven with a humidity of about 25% and a constant temperature of 35°C for 10 h.

(3) Wrap the dehydrated flower with tin foil, and select either one of the following methods for freezing and preservation: one is to freeze and preserve in LN (LN) for storage, and the other is to freeze in LN and then preserve in freezers at −80°C.

(4) Put the preserved flowers into a container filled with enough dry ice, and transport them to the hybridization site by express delivery.

(5) Thaw the foil-wrapped flowers at room temperature for pollination application.

## Data Availability Statement

The original contributions presented in the study are included in the article/supplementary material, further inquiries can be directed to the corresponding authors.

## Author Contributions

XL, HJ, and LZ conducted the major experimental works. XL and YS joined the experiments. JZ, SS, and HY provided advice and technical support. HJ, XYL, TH, JZ, LZ, and ES wrote the manuscript. TH and WL coordinated and supervised the project. All authors read and approved the final manuscript.

## Funding

This work was supported by the Key-Area Research and Development Program of Guangdong Province (2020B020220008), the China Agriculture Research System (CARS-04), and the Chinese Academy of Agricultural Sciences Innovation Project.

## Conflict of Interest

The authors declare that the research was conducted in the absence of any commercial or financial relationships that could be construed as a potential conflict of interest.

## Publisher's Note

All claims expressed in this article are solely those of the authors and do not necessarily represent those of their affiliated organizations, or those of the publisher, the editors and the reviewers. Any product that may be evaluated in this article, or claim that may be made by its manufacturer, is not guaranteed or endorsed by the publisher.
